# Synthesis and crystal structure of a new coordination polymer based on lanthanum and 1,4-phenyl­enedi­acetate ligands

**DOI:** 10.1107/S2056989019002378

**Published:** 2019-02-22

**Authors:** Magatte Camara, Insa Badiane, Mamoudou Diallo, Carole Daiguebonne, Olivier Guillou

**Affiliations:** a Université Assane Seck de Ziguinchor, LCPM-Groupe Matériaux Inorganiques Chimie Douce et Cristallographie, BP 523 Ziguinchor, Senegal; b Université de Rennes, INSA Rennes, CNRS UMR 6226, Institut des Sciences Chimiques de Rennes, F-35708 Rennes, France

**Keywords:** lanthanum ion, coordination polymer, crystal structure

## Abstract

Reaction in gel between the sodium salt of 1,4-phenyl­enedi­acetic acid (Na_2_C_10_O_4_H_8_–Na_2_ *p*-pda) and lanthanum chloride yields single crystals of the three-dimensional coordination polymer poly[[tetra­aqua­tris­(μ-1,4-phenyl­enedi­acetato)­dilanthanum(III)] octa­hydrate], {[La_2_(C_10_H_8_O_4_)_3_(H_2_O)_4_]·8H_2_O}_∞_. The La^III^ coordination polyhedron can be described as a slightly distorted monocapped square anti­prism. One of the two *p*-pda^2−^ ligands is bound to four La^III^ ions and the other to two La^III^ ions. Each La^III^ atom is coordinated by five ligands, thereby generating a metal–organic framework with potential porosity properties.

## Abstract 2

The crystalline structure of a new three-dimensional coordination polymer based on La^III^ and 1,4-phenyl­enedi­acetate ligands is described.

## Chemical context   

In recent years, one of the most important fields of research in coordination chemistry and crystal engineering has been the design of metal–organic frameworks (MOFs), because of their intriguing network topologies and possible applications in gas storage (Eddaoudi *et al.*, 2002 ▸; Reneike *et al.*, 1999 ▸; Luo *et al.*, 2011*a* ▸,*b* ▸; Kustaryono *et al.*, 2010 ▸), catalysis (Lee *et al.*, 2009 ▸), separation (Hamon *et al.*, 2009 ▸), luminescence (Cui *et al.*, 2012 ▸; Daiguebonne *et al.*, 2008 ▸; Binnemans, 2009 ▸;) and mol­ecular magnetism (Calvez *et al.*, 2008 ▸; Sessoli *et al.*, 2009 ▸). Our group has been involved in this field for more than a decade (Freslon *et al.*, 2014 ▸; Fan *et al.*, 2014 ▸; Luo *et al.*, 2011*a* ▸,*b* ▸; Badiane *et al.*, 2017*a* ▸,*b* ▸). The search for new ligands that can lead to new structural networks and/or new physical properties is a continuous concern (Qiu *et al.*, 2007 ▸; Fan *et al.*, 2015 ▸).

For the synthesis of MOFs, usually two complementary mol­ecular precursors, a cation with vacant coordination sites and a bridging anion, are used to form the coordination polymer. This procedure offers the prospect of rationally designing extended solids with inter­esting properties. Most of the organic ligands used in MOF chemistry are rigid aromatic carboxyl­ates (Luo *et al.*, 2007 ▸; Huang *et al.*, 2009 ▸). Compared to the rigid ligands, using flexible ligands such as 1,2- (Xin *et al.*, 2011 ▸), 1,3- (Wang *et al.*, 2012 ▸) or 1,4-phenyl­enedi­acetate (Fabelo *et al.*, 2009*a* ▸,*b* ▸) to construct coordination polymers seems to be more difficult, and developing synthetic methodologies is still a challenge. However, flexibility of the ligand can promote structural and functional diversity.

Numerous coordination polymers have been reported so far that involve *d*-block metal ions such as Cu^II^ (Singh & Barua, 2009 ▸; Fabelo *et al.*, 2009*a* ▸,*b* ▸; Chen *et al.*, 2010*a* ▸,*b* ▸,*c* ▸), Zn^II^ (Singh & Barua, 2009 ▸), Cd^II^ (Chen *et al.*, 2010*a* ▸,*b* ▸,*c* ▸; Singh & Barua, 2009 ▸; Li *et al.*, 2009 ▸), Mn^II^ (Singh & Barua, 2009 ▸; Chen *et al.*, 2010*a* ▸,*b* ▸,*c* ▸, Co^II^ (Fabelo *et al.*, 2009*a* ▸,*b* ▸; Chen *et al.*, 2010*a* ▸,*b* ▸,*c* ▸; Uebler & LaDuca, 2012 ▸; Li *et al.*, 2009 ▸) and Ni^II^ (Chen *et al.*, 2010*a* ▸,*b* ▸,*c* ▸; Uebler & LaDuca, 2012 ▸; Li *et al.*, 2009 ▸). Lanthan­ide(III) ions have higher and variable coordination numbers (generally between 7 and 12) and incorporate in addition, apart from the main ligands, ancillary ligands such as water mol­ecules into the lanthanide coordination sphere. A large number of studies have been reported on lanthanide coordination polymers based on 1,4-phenyl­enedi­acetic acid (Singh & Barua, 2009 ▸; Fabelo *et al.*, 2009*a* ▸,*b* ▸; Chen *et al.*, 2010*a* ▸,*b* ▸,*c* ▸; Uebler & LaDuca, 2012 ▸; Li *et al.*, 2009 ▸; Rusinek *et al.*, 2013 ▸) as well as on other isomers of this acid such as 1,2- (Badiane *et al.*, 2017*a* ▸,*b* ▸; Xin *et al.*, 2011 ▸) and 1,3-phenyl­enedi­acetic acid (Wang *et al.*, 2012 ▸), and most of them tend to make porous materials through solvothermal synthesis.

**Figure 1:**
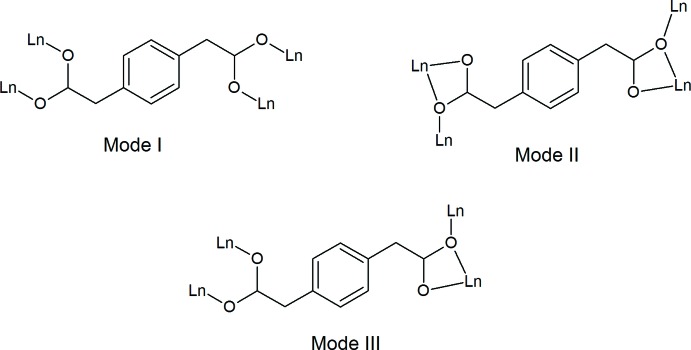
Bonding modes in lanthanide-containing coordination polymers with 1,4-phenyl­enedi­acetate ligands (*p*-pda^2−^) reported in the literature to date.

Isomers of phenyl­enedi­acetic acid are flexible ligands and can therefore adopt different conformations in the crystal structure. 1,4-Phenyl­endi­acetic acid is used as a readily available ligand that can coordinate two or more metal ions in bridging-mode, forming extended mol­ecular networks (Pan *et al.*, 2003 ▸; Chen *et al.*, 2010*a* ▸,*b* ▸,*c* ▸). The different coordination modes (Chen *et al.*, 2010*a* ▸,*b* ▸,*c* ▸; Rusinek *et al.*, 2013 ▸; Ren *et al.*, 2011 ▸; Pan *et al.*, 2003 ▸; Singha *et al.*, 2014 ▸; Singha *et al.*, 2015 ▸) of the ligand with lanthanide ions that have been reported to date are shown in Fig. 1 ▸.

In this paper we report the synthesis and the crystal structure of a new coordination polymer with chemical formula [La_2_(*p*-pda)_3_(H_2_O)_4_·8H_2_O]_∞_.
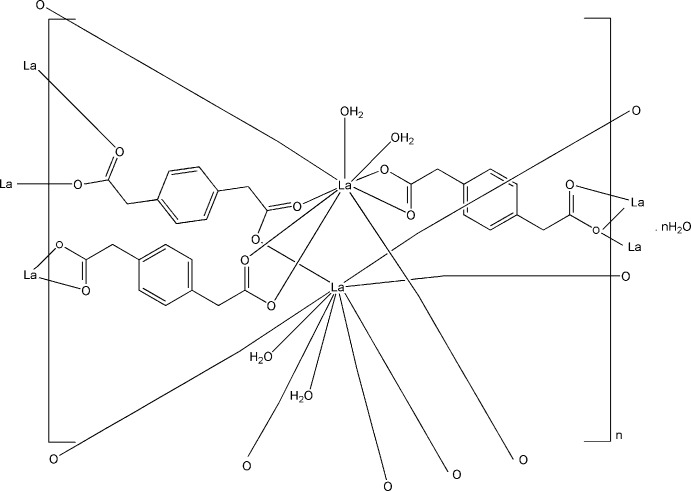


## Structural commentary   

**Figure 2:**
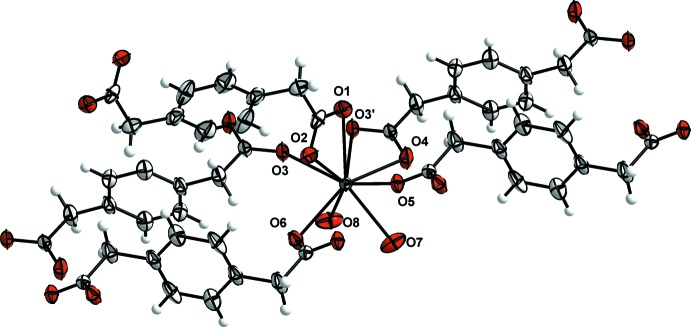
Coordination environment of La^3+^ in [La_2_(*p*-pda)_3_(H_2_O)_4_·8H_2_O]_∞_. Symmetry code: (′) 2 − *x*, 1 − *y*, 1 − *z.* Hydrogen atoms of the water mol­ecules have been omitted for clarity.

**Figure 3:**
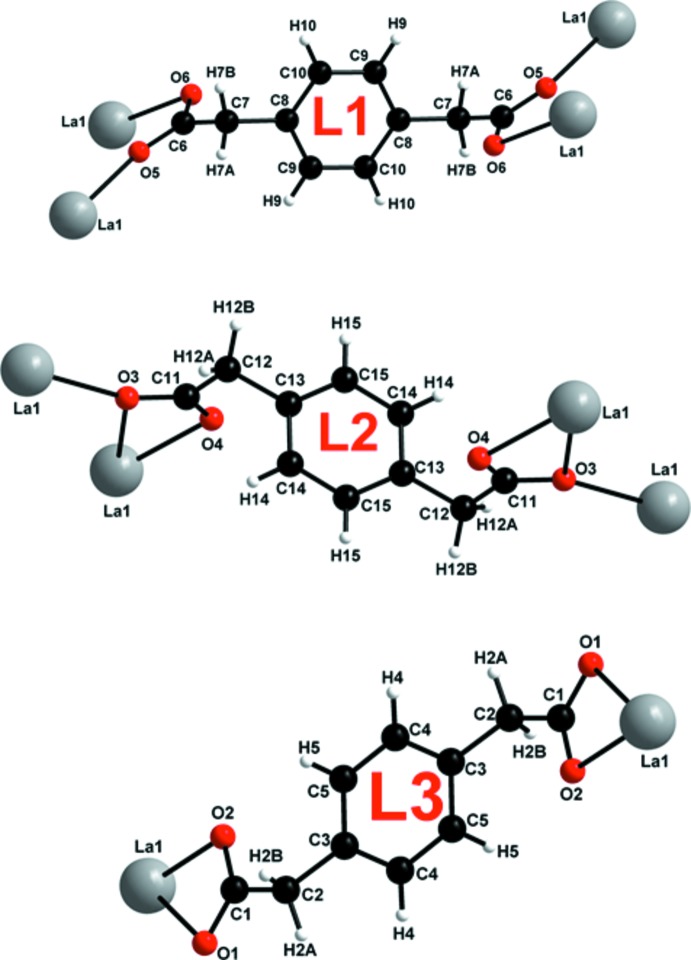
Coordination modes of ligand **L1** (μ-4 bis-bidentate mode: (η^1^-η^1^-μ_2_)-(η^1^-η^1^-μ_2_)-μ_4_), **L2** (μ-4 bis-tridentate bridging and chelating mode: (η^2^-η^1^-μ_2_)-(η^2^-η^1^-μ_2_)-μ_4_) and **L3** (μ-2 bis-bidentate-chelating mode: (η^1^-η^1^-μ_1_)-(η^1^-η^1^-μ_1_)-μ_2_)).

The crystallographically independent La^3+^ ion is nona-coordinated by seven oxygen atoms (O1, O2, O3, O4, O5, O6, O3^’^) from five *p*-pda^2−^ ligands and two oxygen atoms (O8 and O7) from the coordinating water mol­ecules (Fig. 2 ▸). The coordination polyhedron can be described as a monocapped distorted square anti­prism with atom O3′ capping the polyhedron [symmetry code: (′) 2 − *x*, 1 − *y*, 1 − *z*]. The two square sides of the anti­prism are formed by atoms O7, O6, O2, O5 and O8, O3, O1, O4, respectively. The dihedral angle between the two faces is 5.21 (9)°. There are three independent ligands: **L1**, **L2** and **L3** (Fig. 3 ▸). The twisted ligand **L3** exhibits a coordination mode that has never previously been observed in lanthanide-based coordination polymers involving the *p*-pda^2−^ ligand.

**Figure 4:**
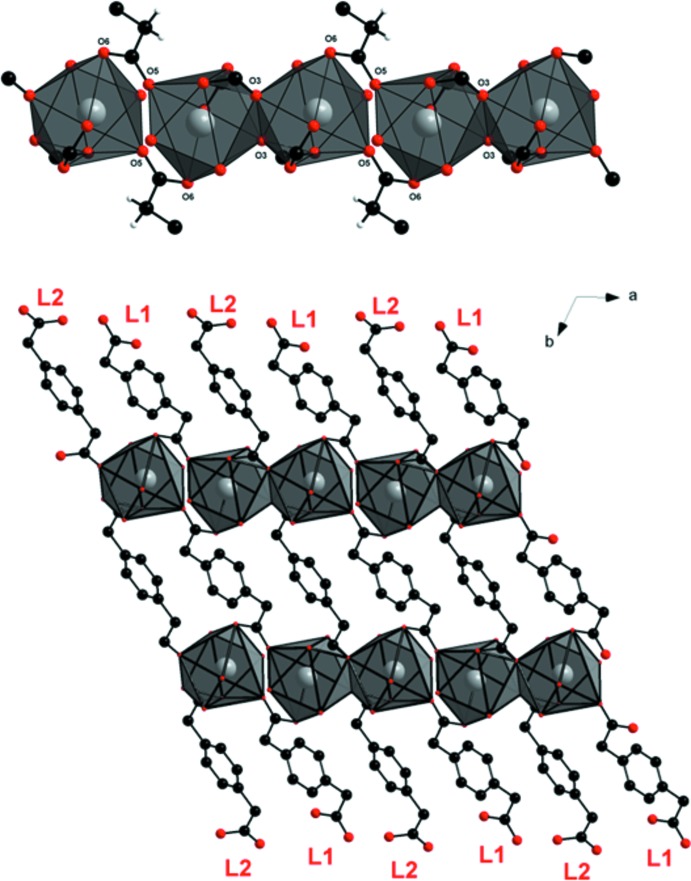
(Top) Projection view of a mol­ecular chain extending parallel to the *a* axis. (Bottom) Projection view along the *c* axis of the of the two-dimensional mol­ecular network of [La_2_(*p*-pda)_3_(H_2_O)_4_·8H_2_O]_∞_. Hydrogen atoms have been omitted for clarity.

**Figure 5:**
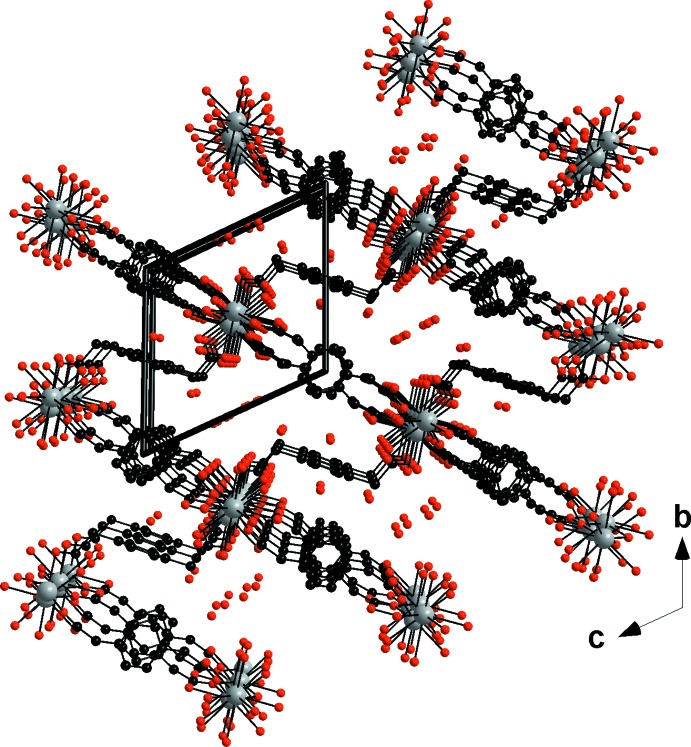
Perspective view along the *a* axis of [La_2_(*p*-pda)_3_(H_2_O)_4_·8H_2_O]_∞_. Hydrogen atoms have been omitted for clarity.

The monocapped square anti­prisms are connected to each other by alternating **L1** bridging carboxyl­ate oxygen atoms (O5 and O6) and edge-sharing polyhedra through **L2** oxygen atoms (O3), forming mol­ecular chains along the *a*-axis direction (Fig. 4 ▸). These chains are connected to each other through ligands **L1** and **L2**, which play the role of spacers, forming mol­ecular layers that extend parallel to the *ab* plane (Fig. 4 ▸). These layers are further connected through the twisted ligand **L3**, leading to a three-dimensional mol­ecular framework (Fig. 5 ▸). Ligand **L3** acts as a spacer between the different polymeric layers because of its *anti–anti* conformation.

**Figure 6:**
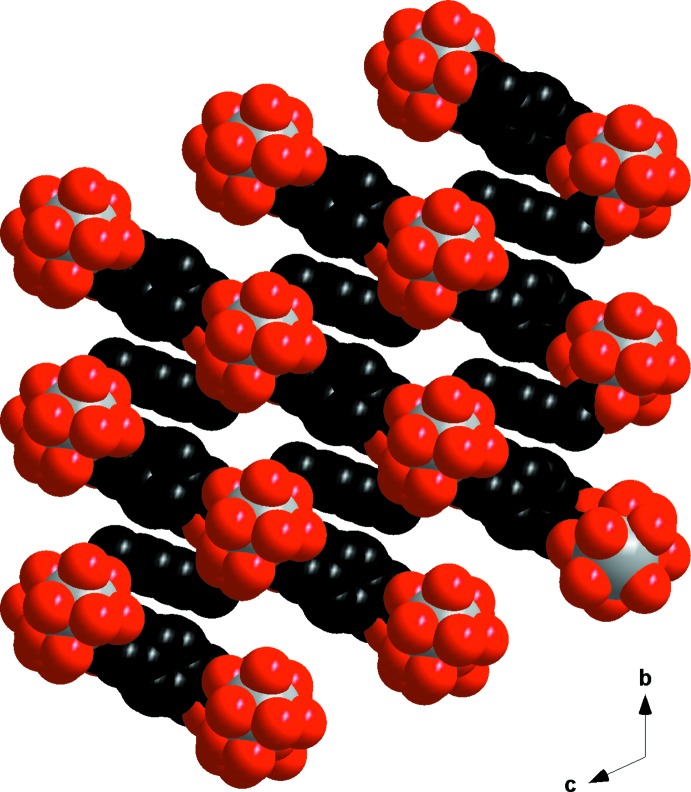
Projection view along the *a* axis of the mol­ecular skeleton of [La_2_(*p*-pda)_3_(H_2_O)_4_·8H_2_O]_∞_ in space-filling mode. Hydrogen atoms and crystallization water mol­ecules have been omitted.

The framework has channels along the *a-*axis direction in which the water mol­ecules of crystallization are located. They are bound to the mol­ecular skeleton *via* a hydrogen-bonded network (Table 1 ▸). As can be seen from Fig. 6 ▸, the three-dimensional crystal structure could potentially present some porosity properties. Indeed, removal of the water mol­ecules of crystallization could create empty channels, as has been reported previously (Kustaryono *et al.*, 2010 ▸; Kerbellec *et al.*, 2008 ▸). For the coordination polymer in this study, the potential porosity is calculated to be 750 (20) m^2^ g^−1^ for N_2_ with a kinetic radius of 1.83 Å. The calculation was performed using a method described elsewhere (Kustaryono *et al.*, 2010 ▸; Kerbellec *et al.*, 2008 ▸).

Other crystal structures of lanthanide coordination polymers with the *p*-pda^2−^ ligand have been reported previously. This series of compounds, first described by Pan *et al.* (2003 ▸) has been widely studied because of potential applications in various fields such as explosives detection (Singha *et al.*, 2014 ▸, 2015 ▸), gas sorption (Pan *et al.*, 2003 ▸) or catalysis (Ren *et al.*, 2011 ▸). These compounds, with general chemical formula [*Ln*_2_(*p*-pda)_3_(H_2_O)·2H_2_O]_∞_ with *Ln* = La–Ho have been obtained by hydro­thermal synthesis and therefore present a lower hydration rate and a higher density than [La_2_(*p*-pda)_3_(H_2_O)_4_·8H_2_O]_∞_ {*D*_calc_ = 1871 g cm^−3^ for [*Ln*_2_(p-pda)_3_(H_2_O)·2H_2_O]_∞_}. Their three-dimensional crystal structures can be described on the basis of helicoidal mol­ecular chains linked by *p*-pda^2−^ ligands.

The luminescent and porosity properties of these compounds are inter­esting, which suggests that the physical properties of compounds isostructural to [La_2_(*p*-pda)_3_(H_2_O)_4_·8H_2_O]_∞_ and involving other lanthanide ions (lanthanum is a diamagnetic non-luminescent ion) would be worth studying. Unfortunately, despite great synthetic efforts, no such compound has been obtained to date.

The compound reported here was obtained by crystallization in a gel (see next section; Luo *et al.*, 2013 ▸), and as such is the first result from our group related to lanthanide-based coordination polymers with 1,4-phenyl­enedi­acetate ligands.

## Synthesis and crystallization   

Lanthanum oxide (La_2_O_3_) was suspended in a small qu­antity of water. The suspension was then brought to about 323 K and concentrated hydro­chloric acid was added dropwise under magnetic stirring, until a clear solution was obtained. The solution was then evaporated to dryness and the resulting solid was dissolved in absolute ethanol for removal of the residual hydro­chloric acid. Crystallization of the salt was then obtained by adding diethyl ether (Et_2_O). The obtained microcrystalline solid was filtered and dried in the open air. The product LaCl_3_·7H_2_O was obtained in close to 100% yield.

1,4-Phenyl­enedi­acetic acid, H_2_(p-pda), was purchased from Sigma–Aldrich and used without further purification. Its disodium salt was prepared by addition of two equivalents of sodium hydroxide to a suspension of the acid in de-ionized water. The obtained clear solution was evaporated to dryness and then refluxed in ethanol for one h. Addition of diethyl ether provoked precipitation of Na_2_(*p*-pda) in 90% yield. UV–vis absorption spectrum of a 4.3 × 10 ^−4^ mol L^−1^ aqueous solution of the disodium salt of H_2_(*p*-pda) was measured with a Perkin–Elmer Lambda 650 spectrometer equipped with a 60 mm integrating sphere. It showed a maximum absorption at 225 nm. This short absorption wavelength, compared to other ligands in the literature (Badiane *et al.*, 2017*a* ▸,*b* ▸; Freslon *et al.*, 2016 ▸; Fan *et al.*, 2015 ▸; Badiane *et al.*, 2018 ▸), can be related to the –CH_2_– groups that cut conjugation.

Single crystals of the coordination polymer were obtained by slow diffusion of dilute aqueous solutions of lanthanum chloride (0.25 mmol in 10 mL) and of the sodium salt of *para*-phenyl­enedi­acetate (0.25 mmol in 10 mL) through an agar-agar gel in a U-shaped tube. The gel was purchased from Acros Organics and jellified according to established procedures (Henisch, 1988 ▸; Daiguebonne *et al.*, 2003 ▸). After several weeks, prismatic single crystals were obtained.

## Refinement   

Crystal data, data collection and structure refinement details are summarized in Table 2 ▸. Hydrogen atoms bound to the organic ligands were placed at idealized positions (C—H = 0.93–0.97 Å) and refined as riding with *U*_iso_(H) = 1.2*U*_eq_(C). The water hydrogen atoms were localized and constrained. The thermal agitation of the two water mol­ecules of crystallization was constrained. In order to stabilize the refinement several restraints (DANG, DFIX) were used for the hydrogen atoms bound to water oxygens.

## Supplementary Material

Crystal structure: contains datablock(s) global, I. DOI: 10.1107/S2056989019002378/vn2143sup1.cif

Structure factors: contains datablock(s) I. DOI: 10.1107/S2056989019002378/vn2143Isup2.hkl

Table 2. Valence angles and torsion angles around the ligands. DOI: 10.1107/S2056989019002378/vn2143sup3.pdf

CCDC reference: 1875083

Additional supporting information:  crystallographic information; 3D view; checkCIF report

## Figures and Tables

**Table 1:** Hydrogen-bond geometry (Å, °)

*D*—H⋯*A*	*D*—H	H⋯*A*	*D*⋯*A*	*D*—H⋯*A*
O*W*1—H*W*1*A*⋯O*W*1^iii^	0.87 (9)	2.40 (11)	3.067 (13)	133 (9)
O*W*1—H*W*1*B*⋯O*W*4^iv^	0.89 (9)	2.54 (10)	3.298 (11)	145 (7)
O*W*2—H*W*2*A*⋯O4^i^	0.82 (6)	2.10 (6)	2.895 (5)	164 (6)
O*W*2—H*W*2*B*⋯O*W*4^v^	0.82 (6)	2.20 (5)	2.855 (8)	137 (5)
O*W*3—H*W*3*A*⋯O6^i^	0.82 (8)	2.02 (8)	2.780 (8)	154 (7)
O*W*3—H*W*3*B*⋯O*W*1^iii^	0.81 (7)	2.40 (8)	3.162 (11)	156 (8)
O*W*4—H*W*4*A*⋯O*W*2^vi^	0.81 (10)	2.49 (9)	2.855 (8)	109 (9)
O7—H7*A*⋯O2^i^	0.82 (4)	1.95 (4)	2.741 (5)	161 (5)
O7—H7*B*⋯O*W*4	0.81 (5)	2.03 (5)	2.800 (9)	160 (5)
O*W*4—H*W*4*B*⋯O*W*3^vii^	0.84 (9)	2.11 (10)	2.824 (11)	143 (8)
O8—H8*A*⋯O*W*3^i^	0.82 (4)	2.38 (4)	3.175 (8)	165 (4)
O8—H8*B*⋯O1^ii^	0.83 (4)	1.92 (4)	2.725 (5)	163 (5)
C7—H7*D*⋯O4^i^	0.97	2.54	3.442 (6)	154
C12—H12*B*⋯O6^ii^	0.97	2.51	3.406 (6)	154

Symmetry codes: (i) 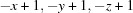; (ii) 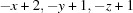; (iii) 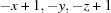; (iv) 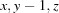; (v) 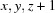; (vi) 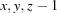; (vii) 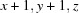.

**Table 2:** Experimental details

Crystal data	
Chemical formula	[La_2_(C_10_H_8_O_4_)_3_(H_2_O)_4_]·8H_2_O
*M*_r_	1070.05
Crystal system, space group	Triclinic, *P* 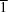
Temperature (K)	293
*a*, *b*, *c* (Å)	9.1197 (2), 11.1231 (2), 11.9434 (2)
α, β, γ (°)	107.049 (1), 107.729 (1), 106.622 (1)
*V* (Å^3^)	1005.21 (3)
*Z*	1
Radiation type	Mo *K*α
μ (mm^−1^)	2.18
Crystal size (mm)	0.08 × 0.06 × 0.05

Data collection	
Diffractometer	Nonius KappaCCD
No. of measured, independent and observed [*I* > 2σ(*I*)] reflections	4588, 4588, 3751
*R*_int_	0.045
(sin θ/λ)_max_ (Å^−1^)	0.649

Refinement	
*R*[*F* ^2^ > 2σ(*F* ^2^)], *wR*(*F* ^2^), *S*	0.030, 0.068, 1.03
No. of reflections	4588
No. of parameters	283
No. of restraints	18
H-atom treatment	H atoms treated by a mixture of independent and constrained refinement
Δρ_max_, Δρ_min_ (e Å^−3^)	1.76, −0.65

Computer programs: *COLLECT* (Bruker, 2004), *DENZO* and *SCALEPACK* (Otwinowski & Minor, 1997 ▸), *SHELXT* (Sheldrick, 2015*a* ▸), *SHELXL* (Sheldrick, 2015*b* ▸), *DIAMOND* (Brandenburg, 2001 ▸), *WinGX* (Farrugia, 2012 ▸).

## supplementary crystallographic information

### Crystal data

**Table:**

[La_2_(C_10_H_8_O_4_)_3_(H_2_O)_4_]·8H_2_O	*Z* = 1
*M**_r_* = 1070.05	*F*(000) = 534
Triclinic, *P*1	*D*_x_ = 1.768 Mg m^−^^3^
Hall symbol: -P 1	Mo *K*α radiation, λ = 0.71073 Å
*a* = 9.1197 (2) Å	Cell parameters from 15558 reflections
*b* = 11.1231 (2) Å	θ = 2.9–27.5°
*c* = 11.9434 (2) Å	µ = 2.18 mm^−^^1^
α = 107.049 (1)°	*T* = 293 K
β = 107.729 (1)°	Prism, colorless
γ = 106.622 (1)°	0.08 × 0.06 × 0.05 mm
*V* = 1005.21 (3) Å^3^	

### Data collection

**Table:**

Nonius KappaCCD diffractometer	3751 reflections with *I* > 2σ(*I*)
Radiation source: Enraf Nonius FR590	*R*_int_ = 0.045
Horizonally mounted graphite crystal monochromator	θ_max_ = 27.5°, θ_min_ = 3.6°
Detector resolution: 9 pixels mm^-1^	*h* = −10→11
CCD rotation images, thick slices scans	*k* = −14→14
4588 measured reflections	*l* = −15→15
4588 independent reflections	

### Refinement

**Table:**

Refinement on *F*^2^	Primary atom site location: dual
Least-squares matrix: full	Secondary atom site location: dual
*R*[*F*^2^ > 2σ(*F*^2^)] = 0.030	Hydrogen site location: mixed
*wR*(*F*^2^) = 0.068	H atoms treated by a mixture of independent and constrained refinement
*S* = 1.03	*w* = 1/[σ^2^(*F*_o_^2^) + (0.0308*P*)^2^] where *P* = (*F*_o_^2^ + 2*F*_c_^2^)/3
4588 reflections	(Δ/σ)_max_ = 0.001
283 parameters	Δρ_max_ = 1.76 e Å^−^^3^
18 restraints	Δρ_min_ = −0.65 e Å^−^^3^
0 constraints	

### Special details

**Table:**

Geometry. All esds (except the esd in the dihedral angle between two l.s. planes) are estimated using the full covariance matrix. The cell esds are taken into account individually in the estimation of esds in distances, angles and torsion angles; correlations between esds in cell parameters are only used when they are defined by crystal symmetry. An approximate (isotropic) treatment of cell esds is used for estimating esds involving l.s. planes.

### Fractional atomic coordinates and isotropic or equivalent isotropic displacement parameters (Å^2^)

**Table:**

	*x*	*y*	*z*	*U*_iso_*/*U*_eq_	
La1	0.75090 (2)	0.52031 (2)	0.47969 (2)	0.02048 (7)	
O5	0.4472 (3)	0.3677 (2)	0.4069 (2)	0.0332 (6)	
O3	1.0575 (3)	0.5881 (3)	0.6253 (2)	0.0323 (6)	
O1	0.7627 (3)	0.3041 (3)	0.5311 (2)	0.0336 (6)	
O6	0.8146 (3)	0.7579 (3)	0.6435 (3)	0.0362 (6)	
O2	0.7422 (3)	0.4674 (3)	0.6736 (2)	0.0351 (6)	
O4	0.6840 (3)	0.3616 (3)	0.2490 (2)	0.0415 (7)	
O7	0.5690 (4)	0.5882 (4)	0.3233 (3)	0.0486 (8)	
H7A	0.468 (3)	0.558 (4)	0.307 (5)	0.058*	
H7B	0.591 (5)	0.651 (4)	0.302 (5)	0.058*	
O8	0.9316 (4)	0.7051 (3)	0.4326 (4)	0.0503 (8)	
H8A	0.947 (6)	0.785 (3)	0.470 (4)	0.060*	
H8B	1.026 (4)	0.712 (4)	0.435 (5)	0.060*	
C6	0.6834 (4)	0.7426 (3)	0.6621 (3)	0.0260 (7)	
C11	0.8165 (4)	0.3436 (3)	0.2659 (3)	0.0261 (7)	
C1	0.7640 (4)	0.3589 (4)	0.6405 (4)	0.0315 (8)	
C13	0.6579 (5)	0.1147 (4)	0.0739 (4)	0.0340 (9)	
C12	0.8247 (5)	0.2382 (4)	0.1565 (4)	0.0390 (9)	
H12A	0.857342	0.281262	0.102898	0.058*	
H12B	0.910563	0.207990	0.192279	0.058*	
C14	0.6017 (5)	0.0190 (4)	0.1196 (4)	0.0427 (10)	
H14	0.669560	0.030195	0.201149	0.051*	
C8	0.8472 (5)	0.9325 (4)	0.8881 (4)	0.0340 (9)	
C10	0.9564 (5)	1.0646 (4)	0.9217 (4)	0.0433 (10)	
H10	0.928413	1.109955	0.869797	0.052*	
C15	0.5547 (5)	0.0946 (4)	−0.0469 (4)	0.0412 (10)	
H15	0.589046	0.157191	−0.080797	0.049*	
C7	0.6832 (5)	0.8578 (4)	0.7675 (4)	0.0445 (11)	
H7C	0.660724	0.923722	0.734480	0.067*	
H7D	0.592152	0.819798	0.789973	0.067*	
C9	0.8927 (6)	0.8687 (4)	0.9685 (4)	0.0461 (11)	
H9	0.820475	0.779508	0.948162	0.055*	
C3	0.8997 (5)	0.3972 (4)	0.8732 (4)	0.0392 (9)	
C4	1.0738 (6)	0.4427 (5)	0.9267 (4)	0.0514 (11)	
H4	1.125405	0.405378	0.878263	0.062*	
C5	0.8281 (6)	0.4561 (5)	0.9477 (4)	0.0501 (11)	
H5	0.711419	0.427472	0.913133	0.060*	
C2	0.7931 (6)	0.2928 (4)	0.7347 (4)	0.0460 (10)	
H2A	0.684726	0.235879	0.727251	0.069*	
H2B	0.848359	0.233001	0.711571	0.069*	
OW1	0.6313 (10)	0.0072 (5)	0.4440 (6)	0.1327 (17)	
HW1A	0.589 (11)	−0.025 (9)	0.491 (8)	0.159*	
HW1B	0.639 (13)	−0.072 (6)	0.411 (8)	0.159*	
OW2	0.5351 (5)	0.6185 (4)	0.9718 (4)	0.0741 (11)	
HW2A	0.476 (7)	0.639 (6)	0.920 (5)	0.111*	
HW2B	0.622 (6)	0.678 (5)	1.033 (5)	0.111*	
OW3	0.0357 (9)	0.0056 (6)	0.3870 (6)	0.1327 (17)	
HW3A	0.059 (10)	0.059 (7)	0.354 (7)	0.159*	
HW3B	0.122 (7)	0.023 (10)	0.447 (6)	0.159*	
OW4	0.7105 (10)	0.7873 (7)	0.2433 (6)	0.154 (3)	
HW4A	0.649 (10)	0.805 (11)	0.192 (8)	0.185*	
HW4B	0.810 (5)	0.831 (11)	0.255 (10)	0.185*	

### Atomic displacement parameters (Å^2^)

**Table:**

	*U*^11^	*U*^22^	*U*^33^	*U*^12^	*U*^13^	*U*^23^
La1	0.01729 (10)	0.02001 (10)	0.01850 (10)	0.00633 (7)	0.00482 (7)	0.00498 (7)
O5	0.0242 (13)	0.0258 (13)	0.0312 (13)	0.0032 (10)	0.0034 (11)	0.0040 (11)
O3	0.0227 (13)	0.0340 (14)	0.0229 (12)	0.0074 (11)	0.0027 (10)	0.0011 (11)
O1	0.0352 (14)	0.0353 (14)	0.0339 (14)	0.0161 (12)	0.0168 (12)	0.0155 (12)
O6	0.0247 (13)	0.0283 (13)	0.0415 (15)	0.0089 (10)	0.0129 (12)	−0.0005 (11)
O2	0.0403 (15)	0.0427 (15)	0.0332 (14)	0.0254 (13)	0.0173 (12)	0.0200 (12)
O4	0.0267 (14)	0.0515 (17)	0.0253 (13)	0.0175 (12)	0.0040 (11)	−0.0052 (12)
O7	0.0425 (17)	0.075 (2)	0.067 (2)	0.0390 (17)	0.0345 (17)	0.0532 (18)
O8	0.0456 (18)	0.0523 (18)	0.088 (2)	0.0315 (16)	0.0434 (18)	0.0469 (19)
C6	0.0228 (17)	0.0219 (17)	0.0260 (17)	0.0100 (14)	0.0051 (14)	0.0052 (14)
C11	0.0187 (16)	0.0250 (17)	0.0236 (16)	0.0060 (13)	0.0062 (14)	0.0018 (14)
C1	0.0184 (17)	0.041 (2)	0.0332 (19)	0.0099 (15)	0.0083 (15)	0.0177 (17)
C13	0.0261 (19)	0.030 (2)	0.0297 (19)	0.0117 (16)	0.0070 (16)	−0.0041 (16)
C12	0.0233 (18)	0.040 (2)	0.0318 (19)	0.0098 (16)	0.0074 (16)	−0.0057 (17)
C14	0.039 (2)	0.044 (2)	0.028 (2)	0.0198 (19)	0.0002 (18)	0.0047 (18)
C8	0.031 (2)	0.0293 (19)	0.0290 (19)	0.0102 (16)	0.0138 (16)	−0.0032 (16)
C10	0.042 (2)	0.033 (2)	0.037 (2)	0.0058 (18)	0.0114 (19)	0.0059 (18)
C15	0.043 (2)	0.037 (2)	0.031 (2)	0.0133 (19)	0.0076 (18)	0.0078 (17)
C7	0.026 (2)	0.040 (2)	0.043 (2)	0.0127 (17)	0.0102 (18)	−0.0078 (19)
C9	0.042 (2)	0.0225 (19)	0.048 (2)	−0.0033 (17)	0.016 (2)	0.0006 (18)
C3	0.043 (2)	0.044 (2)	0.035 (2)	0.0206 (19)	0.0123 (18)	0.0260 (18)
C4	0.051 (3)	0.073 (3)	0.041 (2)	0.037 (2)	0.022 (2)	0.023 (2)
C5	0.032 (2)	0.077 (3)	0.042 (2)	0.024 (2)	0.0112 (19)	0.027 (2)
C2	0.055 (3)	0.040 (2)	0.039 (2)	0.017 (2)	0.012 (2)	0.0233 (19)
OW1	0.192 (4)	0.069 (2)	0.109 (3)	0.009 (3)	0.079 (3)	0.028 (2)
OW2	0.063 (3)	0.086 (3)	0.074 (3)	0.032 (2)	0.020 (2)	0.043 (2)
OW3	0.192 (4)	0.069 (2)	0.109 (3)	0.009 (3)	0.079 (3)	0.028 (2)
OW4	0.158 (6)	0.115 (4)	0.103 (4)	−0.017 (4)	−0.019 (4)	0.084 (4)

### Geometric parameters (Å, º)

**Table:**

La1—O5	2.507 (2)	C12—H12B	0.9700
La1—O5^i^	2.905 (3)	C14—H14	0.9300
La1—O3^ii^	2.781 (2)	C14—C15^iii^	1.399 (6)
La1—O3	2.545 (2)	C8—C10	1.373 (6)
La1—O1	2.673 (2)	C8—C7	1.509 (5)
La1—O6	2.559 (2)	C8—C9	1.388 (6)
La1—O2	2.569 (2)	C10—H10	0.9300
La1—O4	2.566 (2)	C10—C9^iv^	1.382 (6)
La1—O7	2.543 (3)	C15—H15	0.9300
La1—O8	2.562 (3)	C7—H7C	0.9700
La1—C11	3.066 (3)	C7—H7D	0.9700
La1—C1	2.988 (4)	C9—H9	0.9300
O5—C6^i^	1.255 (4)	C3—C4	1.385 (6)
O3—C11^ii^	1.264 (4)	C3—C5	1.381 (6)
O1—C1	1.264 (4)	C3—C2	1.509 (6)
O6—C6	1.256 (4)	C4—H4	0.9300
O2—C1	1.248 (4)	C4—C5^v^	1.391 (6)
O4—C11	1.246 (4)	C5—H5	0.9300
O7—H7A	0.824 (19)	C2—H2A	0.9700
O7—H7B	0.806 (18)	C2—H2B	0.9700
O8—H8A	0.815 (19)	OW1—HW1A	0.87 (2)
O8—H8B	0.833 (19)	OW1—HW1B	0.88 (2)
C6—C7	1.512 (5)	OW2—HW2A	0.821 (19)
C11—C12	1.516 (5)	OW2—HW2B	0.81 (2)
C1—C2	1.514 (5)	OW3—HW3A	0.82 (2)
C13—C12	1.507 (5)	OW3—HW3B	0.81 (2)
C13—C14	1.376 (6)	OW4—HW4A	0.81 (2)
C13—C15	1.371 (5)	OW4—HW4B	0.84 (2)
C12—H12A	0.9700		
			
O5—La1—O5^i^	61.48 (9)	C11—O4—La1	101.3 (2)
O5—La1—O3	146.45 (9)	La1—O7—H7A	116 (3)
O5—La1—O3^ii^	118.58 (7)	La1—O7—H7B	133 (3)
O5—La1—O1	75.87 (8)	H7A—O7—H7B	109 (3)
O5—La1—O6	107.86 (8)	La1—O8—H8A	118 (3)
O5—La1—O2	75.29 (8)	La1—O8—H8B	122 (3)
O5—La1—O4	80.33 (8)	H8A—O8—H8B	104 (3)
O5—La1—O7	71.93 (9)	O5^i^—C6—La1	68.28 (18)
O5—La1—O8	140.13 (9)	O5^i^—C6—O6	120.2 (3)
O5^i^—La1—C11	156.28 (8)	O5^i^—C6—C7	120.1 (3)
O5—La1—C11	98.48 (8)	O6—C6—La1	52.33 (16)
O5—La1—C1	75.84 (9)	O6—C6—C7	119.7 (3)
O5^i^—La1—C1	88.69 (9)	C7—C6—La1	169.6 (2)
O3—La1—O5^i^	118.22 (7)	O3^ii^—C11—La1	65.08 (17)
O3^ii^—La1—O5^i^	179.00 (7)	O3^ii^—C11—C12	120.3 (3)
O3—La1—O3^ii^	61.11 (8)	O4—C11—La1	55.16 (17)
O3—La1—O1	73.20 (8)	O4—C11—O3^ii^	119.9 (3)
O3—La1—O6	80.93 (8)	O4—C11—C12	119.8 (3)
O3—La1—O2	74.87 (8)	C12—C11—La1	171.2 (2)
O3—La1—O4	108.82 (8)	O1—C1—La1	63.39 (19)
O3—La1—O8	73.39 (9)	O1—C1—C2	119.8 (3)
O3^ii^—La1—C11	24.36 (8)	O2—C1—La1	58.58 (18)
O3—La1—C11	85.47 (8)	O2—C1—O1	121.5 (3)
O3—La1—C1	70.64 (9)	O2—C1—C2	118.7 (3)
O3^ii^—La1—C1	90.37 (9)	C2—C1—La1	172.4 (3)
O1—La1—O5^i^	109.68 (7)	C14—C13—C12	120.5 (4)
O1—La1—O3^ii^	69.49 (8)	C15—C13—C12	122.1 (4)
O1—La1—C11	74.29 (9)	C15—C13—C14	117.4 (4)
O1—La1—C1	25.01 (9)	C11—C12—H12A	109.2
O6—La1—O5^i^	46.41 (7)	C11—C12—H12B	109.2
O6—La1—O3^ii^	133.56 (8)	C13—C12—C11	112.0 (3)
O6—La1—O1	126.21 (9)	C13—C12—H12A	109.2
O6—La1—O2	78.86 (9)	C13—C12—H12B	109.2
O6—La1—O4	149.49 (10)	H12A—C12—H12B	107.9
O6—La1—O8	71.55 (10)	C13—C14—H14	119.1
O6—La1—C11	149.62 (9)	C13—C14—C15^iii^	121.8 (4)
O6—La1—C1	101.90 (10)	C15^iii^—C14—H14	119.1
O2—La1—O5^i^	66.57 (8)	C10—C8—C7	121.6 (4)
O2—La1—O3^ii^	112.44 (8)	C10—C8—C9	117.8 (4)
O2—La1—O1	49.39 (8)	C9—C8—C7	120.6 (4)
O2—La1—C11	123.42 (9)	C8—C10—H10	119.5
O2—La1—C1	24.50 (9)	C8—C10—C9^iv^	121.1 (4)
O4—La1—O5^i^	132.95 (7)	C9^iv^—C10—H10	119.5
O4—La1—O3^ii^	47.75 (7)	C13—C15—C14^iii^	120.7 (4)
O4—La1—O1	84.13 (9)	C13—C15—H15	119.6
O4—La1—O2	131.27 (9)	C14^iii^—C15—H15	119.6
O4—La1—C11	23.49 (8)	C6—C7—H7C	108.9
O4—La1—C1	108.61 (10)	C6—C7—H7D	108.9
O7—La1—O5^i^	70.81 (9)	C8—C7—C6	113.4 (3)
O7—La1—O3	141.53 (10)	C8—C7—H7C	108.9
O7—La1—O3^ii^	110.19 (9)	C8—C7—H7D	108.9
O7—La1—O1	142.43 (10)	H7C—C7—H7D	107.7
O7—La1—O6	82.59 (10)	C8—C9—H9	119.4
O7—La1—O2	134.87 (9)	C10^iv^—C9—C8	121.1 (4)
O7—La1—O4	71.95 (10)	C10^iv^—C9—H9	119.4
O7—La1—O8	68.46 (10)	C4—C3—C2	120.7 (4)
O7—La1—C11	91.69 (10)	C5—C3—C4	118.0 (4)
O7—La1—C1	147.21 (10)	C5—C3—C2	121.2 (4)
O8—La1—O5^i^	108.04 (8)	C3—C4—H4	119.8
O8—La1—O3^ii^	72.59 (9)	C3—C4—C5^v^	120.4 (4)
O8—La1—O1	138.11 (8)	C5^v^—C4—H4	119.8
O8—La1—O2	139.28 (10)	C3—C5—C4^v^	121.6 (4)
O8—La1—O4	83.41 (11)	C3—C5—H5	119.2
O8—La1—C11	78.57 (10)	C4^v^—C5—H5	119.2
O8—La1—C1	144.03 (10)	C1—C2—H2A	109.0
C1—La1—C11	98.95 (10)	C1—C2—H2B	109.0
La1—O5—La1^i^	118.52 (9)	C3—C2—C1	112.8 (3)
C6^i^—O5—La1^i^	88.1 (2)	C3—C2—H2A	109.0
C6^i^—O5—La1	153.4 (2)	C3—C2—H2B	109.0
La1—O3—La1^ii^	118.89 (8)	H2A—C2—H2B	107.8
C11^ii^—O3—La1	150.5 (2)	HW1A—OW1—HW1B	88 (7)
C11^ii^—O3—La1^ii^	90.56 (19)	HW2A—OW2—HW2B	121 (5)
C1—O1—La1	91.6 (2)	HW3A—OW3—HW3B	108 (4)
C6—O6—La1	104.8 (2)	HW4A—OW4—HW4B	107 (4)
C1—O2—La1	96.9 (2)		

Symmetry codes: (i) −*x*+1, −*y*+1, −*z*+1; (ii) −*x*+2, −*y*+1, −*z*+1; (iii) −*x*+1, −*y*, −*z*; (iv) −*x*+2, −*y*+2, −*z*+2; (v) −*x*+2, −*y*+1, −*z*+2.

### Hydrogen-bond geometry (Å, º)

**Table:**

*D*—H···*A*	*D*—H	H···*A*	*D*···*A*	*D*—H···*A*
O*W*1—H*W*1*A*···O*W*1^vi^	0.87 (9)	2.40 (11)	3.067 (13)	133 (9)
O*W*1—H*W*1*B*···O*W*4^vii^	0.89 (9)	2.54 (10)	3.298 (11)	145 (7)
O*W*2—H*W*2*A*···O4^i^	0.82 (6)	2.10 (6)	2.895 (5)	164 (6)
O*W*2—H*W*2*B*···O*W*4^viii^	0.82 (6)	2.20 (5)	2.855 (8)	137 (5)
O*W*3—H*W*3*A*···O6^i^	0.82 (8)	2.02 (8)	2.780 (8)	154 (7)
O*W*3—H*W*3*B*···O*W*1^vi^	0.81 (7)	2.40 (8)	3.162 (11)	156 (8)
O*W*4—H*W*4*A*···O*W*2^ix^	0.81 (10)	2.49 (9)	2.855 (8)	109 (9)
O7—H7*A*···O2^i^	0.82 (4)	1.95 (4)	2.741 (5)	161 (5)
O7—H7*B*···O*W*4	0.81 (5)	2.03 (5)	2.800 (9)	160 (5)
O*W*4—H*W*4*B*···O*W*3^x^	0.84 (9)	2.11 (10)	2.824 (11)	143 (8)
O8—H8*A*···O*W*3^i^	0.82 (4)	2.38 (4)	3.175 (8)	165 (4)
O8—H8*B*···O1^ii^	0.83 (4)	1.92 (4)	2.725 (5)	163 (5)
C7—H7*D*···O4^i^	0.97	2.54	3.442 (6)	154
C12—H12*B*···O6^ii^	0.97	2.51	3.406 (6)	154

Symmetry codes: (i) −*x*+1, −*y*+1, −*z*+1; (ii) −*x*+2, −*y*+1, −*z*+1; (vi) −*x*+1, −*y*, −*z*+1; (vii) *x*, *y*−1, *z*; (viii) *x*, *y*, *z*+1; (ix) *x*, *y*, *z*−1; (x) *x*+1, *y*+1, *z*.
