# Supplementary material for: Synthesis and crystal structure of a new coordination polymer based on lanthanum and 1,4-phenyl­enedi­acetate ligands
Source: Acta Crystallogr E Crystallogr Commun. 2019 Feb 22;75(Pt 3):378–82. doi: 10.1107/S2056989019002378 (PMC6399701; doi:10.1107/S2056989019002378)
Supplement: Supplementary file 3 [file e-75-00378-sup3.pdf]

Table 2. Valence angles and torsion angles around the ligands

Atoms	Valence angles	Atoms	Torsion angles
	Ligand **L1**		
C6-O6-La	104.877(246)°	La-O6-C6-C7	172.030(327)°
O6-C6-O5	119.916(416)°	O6-C6-C7-C8	-38.099(588)°
C6-O5-La	153.173(289)°	C6-C7-C8-C9	108.504(526)°
		La-O5-C6-C7	-71.234(590)°
		O5-C6-C7-C8	142.389(427)°
		C6-C7-C8-C10	108.504(526)°
		C6-C7-C8-C9	-71.234(590)°
		C7-C8-C9-C10	179.533(482)°
		C7-C8-C10-C9	-179.530(483)°
	Ligand **L2**		
C11-O3-La	90.402(211)°	La-O4-C11-C12	171.452(310)°
C11-O4-La	101.257(230)°	O4-C11-C12-C13	-34.625(548)°
O3-La-O4	47.735(85)°	La-O3-C11-C12	6.023(760)°
O4-C11-O3	120.144(342)°	O3-C11-C12-C13	144.168(391)°
La-O3-La	118.873(111)°		
	Ligand **L3**		
C1-O2-La	96.896(270)°	La-O2-C1-C2	171.996(377)°
O2-La-O1	49.500(104)°	O2-C1-C2-C3	-37.982(642)°
La-O1-C1	91.484(269)°	La-O1-C1-C2	-172.337(398)°
O1-C1-O2	121.586(414)°	O1-C1-C2-C3	141.746(461)°
